# Physical Preparation in Female Rugby Codes: An Investigation of Current Practices

**DOI:** 10.3389/fspor.2020.584194

**Published:** 2020-11-26

**Authors:** Omar Heyward, Ben Nicholson, Stacey Emmonds, Gregory Roe, Ben Jones

**Affiliations:** ^1^Carnegie Applied Rugby Research (CARR) Centre, Carnegie School of Sport, Institute for Sport, Leeds Beckett University, Leeds, United Kingdom; ^2^Rugby Football Union, London, United Kingdom; ^3^England Performance Unit, Rugby Football League, Leeds, United Kingdom; ^4^Bath Rugby, Bath, United Kingdom; ^5^Leeds Rhinos Rugby League Club, Leeds, United Kingdom; ^6^School of Science and Technology, University of New England, Armidale, NSW, Australia; ^7^Division of Exercise Science and Sports Medicine, Department of Human Biology, Faculty of Health Sciences, The University of Cape Town and the Sports Science Institute of South Africa, Cape Town, South Africa

**Keywords:** performance, strength, conditioning, sport, athlete, women, survey

## Abstract

Female sports have recently seen a dramatic rise in participation and professionalism world-wide. Despite progress, the infrastructure and general sport science provisions in many female sports are behind their male counterparts. From a performance perspective, marked differences in physical and physiological characteristics can be seen between the sexes. Although physical preparation practices for male athletes are known, there are currently no published literature pertaining exclusively to female athletes. This information would provide invaluable data for both the researcher and practitioner alike. This survey therefore aimed to examine current practices utilized in female rugby codes (union, league, and sevens). A questionnaire assessing seasonal physical preparation practices, recovery, monitoring and sport science technology, and unique aspects in female rugby was developed. Thirty-seven physical preparation practitioners (32 males, 5 females) responded to the questionnaire. Most participants (78%) worked with national or regional/state level female athletes. Performance testing was more frequently assessed in the pre- (97%) and in-season (86%), than off-season (23%). Resistance, cardiovascular, sprint and plyometric training, and recovery sessions were all believed to be important to enhancing performance and implemented by most participants (≥ 89%). Sport science technologies were commonly (54%) utilized to inform current practice. Menstrual cycle phase was monitored by 22% of practitioners. The most frequently reported unique considerations in female rugby codes included psycho-social aspects (41%), the menstrual cycle (22%), and physical differences (22%). Practitioners working with female rugby can use the presented data to inform and develop current practices.

## Introduction

Female sports have recently seen a dramatic rise in participation, professionalism, and profiles world-wide. New female sports leagues (e.g., Australian Football League Women's, Football Association Women's Super League, Rugby Football Union's Premier 15's) have been developed which have been vital in improving exposure, professionalism and infrastructure to female athletes. Despite this progress, anecdotal evidence suggests the infrastructure and general sport science provisions for many female athletes are behind that of male athletes. Sportswomen who compete at comparable levels to sportsmen may have less access to sports performance support (e.g., medical and sport science), this may be a result of lower financial investment (International Working Group on Women and Sport WSI, 2007; Fink, 2015). From a performance perspective, marked differences between the sexes can be seen in anthropometric (Quarrie et al., 1995; Brazier et al., 2018; Sella et al., 2019), movement demands (Ball et al., 2019), physical performance (Sella et al., 2019; Owen et al., 2020) and physiological characteristics (Sheel, 2016). Decreased levels of skeletal muscle mass (Abe et al., 2003), lower rates of muscular fatiguability (Hicks et al., 2001), lower maximum velocity, strength and power have all been previously reported (Quarrie et al., 1995; Brazier et al., 2018; Ball et al., 2019) in females. Additionally, female athletes must consider their menstrual and oral contraceptive pill cycles which may influence athletic performance (Elliott-Sale et al., 2020; McNulty et al., 2020). As there is a lack of female-specific sports performance representation in research studies, female-specific research is urgently needed (Emmonds et al., 2019). Given both the aforementioned contextual and biological sex differences, sports performance research involving male participants cannot necessarily be applied to female cohorts (Emmonds et al., 2019).

While an evidence base from research studies is typically slow to emerge, practice may evolve at a faster rate (Coutts, 2016; Jones B. et al., 2017). Within male athlete literature, the strength and conditioning (S&C) practices (e.g., physical testing, speed, and power training) of various sports have been previously described (Ebben and Blackard, 2001; Ebben et al., 2004, 2005; Simenz et al., 2005; Gee et al., 2011; Winwood et al., 2011; Jones T. et al., 2016; Pote and Christie, 2016; Crowley et al., 2018; Robinson et al., 2019). Research describing the S&C practices applied to male athletes found considerable heterogeneity which suggests either, limited consensus on best-practice or that there are multiple methods available to achieve an end-result. Despite this, these studies have provided thorough overviews of S&C practices which have direct application to applied practitioners when developing physical preparation programmes. To date, there is no research describing S&C practices within a female only cohort, which may be different to male athletes.

Rugby codes (league, union, and sevens) are physiologically demanding intermittent contact sports that involve high-intensity movements (e.g., sprinting and tackling) interspersed with low to moderate intensity activities (e.g., jogging) (Read et al., 2018; Whitehead et al., 2018; Ball et al., 2019; Sella et al., 2019; Weaving et al., 2019; Sheppy et al., 2020). Female rugby sevens demonstrates a greater relative distance demand when compared to both female rugby union and league (~80–120 vs. ~75 m·min^−1^) (Ball et al., 2019; Emmonds et al., 2020; Sheppy et al., 2020). Furthermore, although there is minimal female rugby code collision demand literature available, the relative impacts per game in rugby union and league may be greater than in rugby sevens (Suarez-Arrones et al., 2014; Ball et al., 2019; Sella et al., 2019). In line with sport science literature investigating male athletes, an emerging evidence base, quantifying the anthropometric (Gabbett, 2007; Nyberg and Penpraze, 2016; Agar-Newman et al., 2017), physical (Jones B. et al., 2016; Agar-Newman et al., 2017; Clarke et al., 2017) and physiological (Gabbett, 2007; Suarez-Arrones et al., 2012; Clarke et al., 2014; Nyberg and Penpraze, 2016) profiles of female rugby players have supported practitioners by developing their understanding of the requirements in female rugby. Despite the emerging evidence base, no study has reported the S&C practices applied in female rugby.

Physical preparation pertains to all aspects related to physical performance development and requires consideration of contextual factors (e.g., sex, sport, and playing position) influencing injury and performance. Due to the demands of rugby match-play (e.g., repeated collisions and sprints), physical strength, speed and cardiovascular fitness are integral to successful performance (Jones B. et al., 2016; Clarke et al., 2017; Emmonds et al., 2020; Sheppy et al., 2020). These demands may lead to fatigue which is associated with feelings of tiredness, and muscle function decrements (Twist and Highton, 2013). Appropriately timed recovery modalities can enhance physiological and psychological function post-rugby match-play (Tavares et al., 2017). There has been a recent rise in the use of, and research in, sport science monitoring technologies (Cardinale and Varley, 2017). The use of these technologies can assist the practitioner's day-to-day decision making. In order to develop female rugby physical preparation practices, we must initially understand the current landscape. Comprehensive information regarding these practices would be a vital resource for the applied practitioner. This has implications for developing comprehensive physical preparation programmes and continuing professional development to optimize physical performance and decrease injury risk in female rugby. Previous S&C practices research (Ebben and Blackard, 2001; Ebben et al., 2004, 2005; Simenz et al., 2005; Gee et al., 2011; Winwood et al., 2011; Jones T. et al., 2016; Crowley et al., 2018; Robinson et al., 2019) investigating male athletes have not represented holistic approaches to physical preparation, as certain key aspects to physical performance (e.g., recovery and monitoring) have not been explored. Therefore, the aim of this study was to explore a comprehensive approach to physical preparation practices currently utilized in female rugby codes.

## Materials and Methods

### Design of Study

The questionnaire was adapted from a previous survey (Ebben and Blackard, 2001) which has been used extensively to assess S&C practices in team sports (Ebben and Blackard, 2001; Ebben et al., 2004, 2005; Simenz et al., 2005; Jones T. et al., 2016). Adaptation of the original instrument was performed to expand on gaps in questionnaire design by including additional sections (e.g., recovery, monitoring, and sports science technology) which allows a more comprehensive overview of current practices. Pilot testing for both content and face validity was then performed by experienced (>5 years) S&C coaches and research-practitioners (Bolarinwa, 2015; Jones B. et al., 2017). The questionnaire (Data Sheet 1) comprised of seven sections; participant characteristics, pre-season, in-season and off-season physical preparation, recovery, monitoring and sport science technology, and unique aspects in female rugby. Sections on physical preparation seasonal phases (i.e., pre-season, in-season, and off-season) included sub-sections on physical testing, and resistance, cardiovascular, sprint and plyometric training. The self-administered, online questionnaire was circulated, via email, and social media (e.g., Twitter), to participants working within female rugby at any level of competition.

### Participants

Prior to any experimental procedures commencing Leeds Beckett University Research Ethics Committee approved the study (#59730). All participants were informed of the risks and benefits of the study before signing an electronic informed consent form. Participants were included in the study if they provided physical preparation support to female rugby athletes (e.g., S&C coaches), at any level of competition.

### Procedures

The questionnaire was circulated electronically from April to August 2019. Participants provided their education and coaching qualifications prior to engaging in the questionnaire. Follow-up correspondence, via email, and social media, to encourage non-responders to complete the questionnaire was sent out 3 weeks after initial circulation. Responses were collected and analyzed with Qualtrics software (Qualtrics, Provo, USA) and Microsoft Office 365 ProPlus Excel (version 1902; Microsoft Corporation, Redmond, WA, USA). Only completed questionnaires were included in the final analysis.

### Data Analyses

The survey contained fixed and open-ended response questions. Answers to open-ended questions were analyzed according to the inductive, and then deductive content analysis method (Elo and Kyngäs, 2008), as a means of identifying, analyzing and reporting common patterns (main categories) within the data. Content analysis has been performed in related studies (Gee et al., 2011; Jones T. et al., 2016). Inductive content analysis was initially performed by identifying the main categories via familiarization and open coding, grouping, categorization and abstraction of the raw data. When the main categories were developed, a deductive analysis was used to confirm that all raw data categories were represented. Raw data were defined by actual responses of participants. Both inductive and deductive analysis was performed independently by two investigators (OH and BN). As conflicts between the investigators relating to main categories were minor (e.g., word choice), a third investigator was not necessary. Data were presented as frequencies and/or percentages unless otherwise stated.

## Results

### Participant Characteristics

Thirty-seven participants responded to the questionnaire (Table 1). Participant's degrees were obtained in S&C or Sport and Exercise Science-related fields. Participants were accredited by the United Kingdom Strength and Conditioning Association (35%), the Australian Strength and Conditioning Association (27%), the National Academy of Sports Medicine (USA; 8%). Other certifications held by participants included “*Australian Weightlifting Federation*” (“*italicized text*” are direct quotations taken from the questionnaire), “*Certified Physical Preparation Specialist*,” “*EXOS Performance Specialist*” and “*Westside Barbell Accreditation*.” Nineteen percent of participants held dual certifications with professional associations, while 22% were not certified. Participants worked in both a team and individual environment (59%) or only a team environment (41%).

**Table 1:** Participant characteristics.

	**Frequency [n (%)]**
**Sex**	
Female	5 (14)
Male	32 (86)
**Age^a^ in years**	29.4 ± 5.0
**Level of competition**	
International	5 (14)
National	20 (54)
Regional/State	9 (24)
Recreational/Local	3 (8)
**Country of employment**	
United Kingdom and Northern Ireland	22 (59)
Australia	8 (22)
Canada	2 (5)
New Zealand	2 (5)
Spain	1 (3)
France	1 (3)
South Africa	1 (3)
**Rugby codes^b^**	
Rugby union	28 (56)
Rugby sevens	15 (30)
Rugby league	7 (14)
**Formal education**	
Doctorate degree	2 (5)
Master's degree	24 (65)
Bachelor's degree	9 (24)
High school equivalent	2 (5)

a *mean ± SD*.

b *thirty percent of participants reported working across multiple codes. Values might not add up to exactly 100% due to rounding*.

Eight participants identified their roles as “*head*,” “*lead*” or “*senior*” “*athletic performance*,” “*performance*” or “*S*&*C*” coaches. Twenty-four participants identified as S&C coaches, one of which specialized as a “*women's strength and conditioning coach*” and another with a dual sport science and S&C role. Further respondents identified as 1 “*athletic development coach*,” 1 “*performance coach*,” 1 “*performance specialist*,” 1 “*performance sport and fitness officer*,” and 1 “*physical performance coach*.”

### Training Phase Duration and Weekly Micro-Cycles

**Figure 1:**
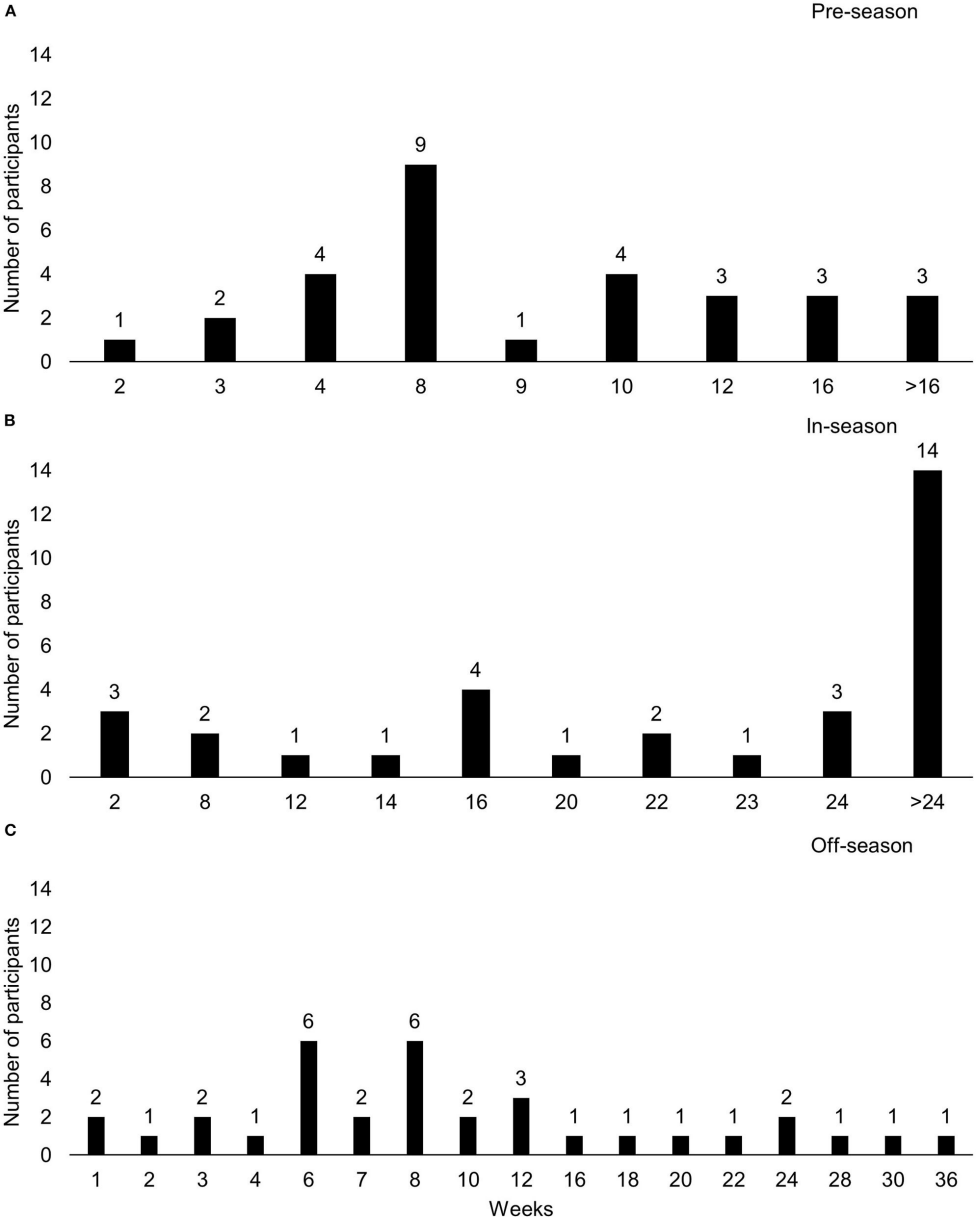
Training phase duration for **(A)** pre-season, **(B)** in-season, and **(C)** off-season.

**Figure 2:**
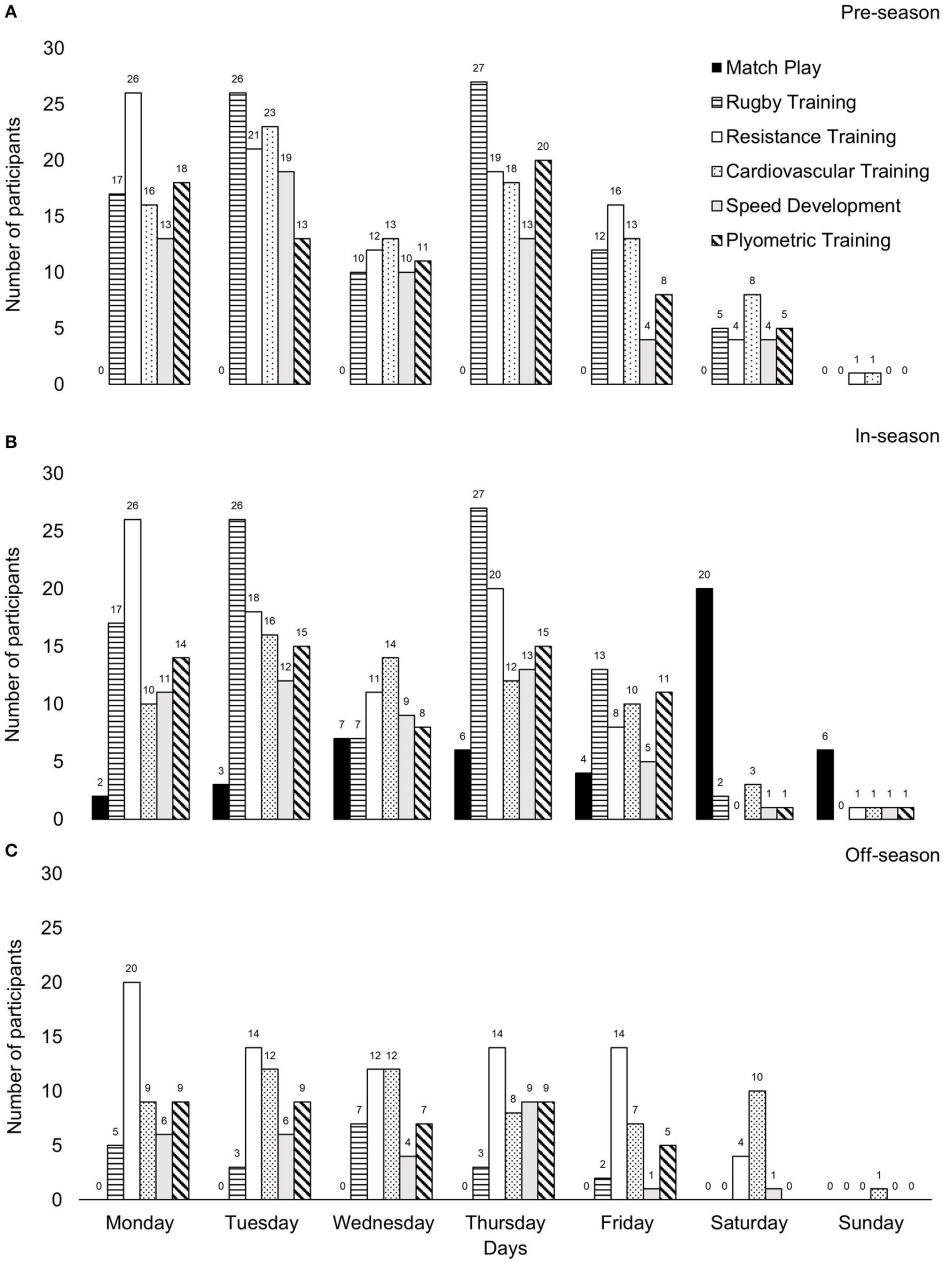
Typical weekly micro-cycles for **(A)** pre-season, **(B)** in-season, and **(C)** off-season.

Pre-, in- and off-season durations are depicted in Figure 1 and typical pre-, in-, and off-season training micro-cycles are depicted in Figure 2.

### Physical Performance Testing

Participants conducted physical performance testing during pre- (97%), in- (86%), and off-season (23%) phases. Responses for non-inclusion of physical performance testing were content analyzed and resulted in three main categories: (a) logistics, (b) miscellaneous, and (c) recovery focus (Table 2).

**Table 2:** Non-inclusion of physical performance testing.

**Main category**	**No. of responses**	**Select raw data representing responses to this question**
Logistics	20	Contact with players is minimal and do not have the resources to manage this in the off season. During off-season players return to their local clubs or other sports.
Miscellaneous^*^	6	Women from 16 to 30+ [years of age] at all different levels of experience, inconsistently turning up, and some do not know how to play the game properly yet, whilst some play [at the international level]. Not worth taking a session away. Due to the stigma and anxious feelings “testing” promotes I don't really see it was a must during the competitive season.
Recovery focus	4	Focus is on mental and physical recovery. Primarily for a psychological break for the athletes.

* *answers that could not be associated with any of the broad identified themes*.

**Figure 3:**
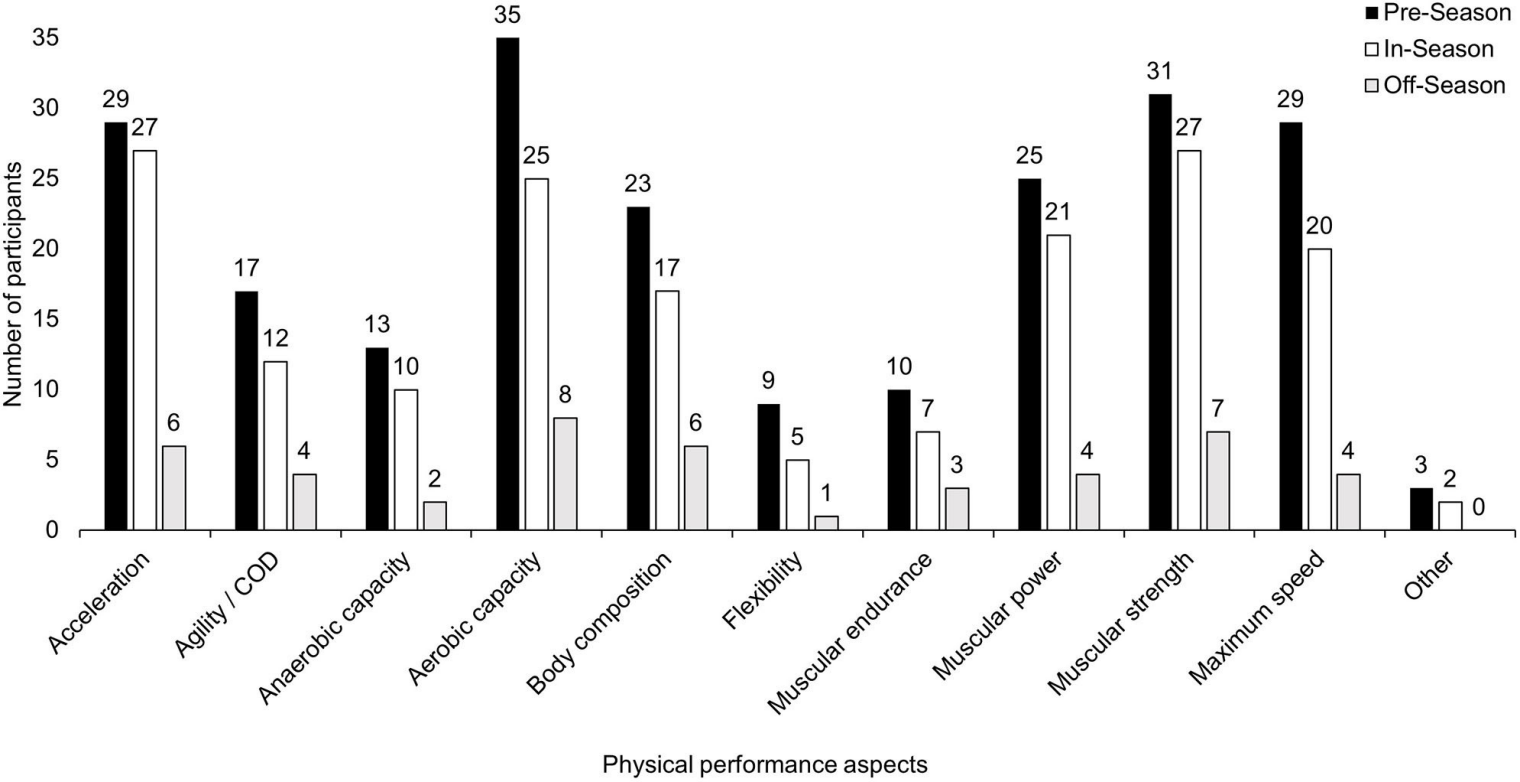
Aspects of physical performance tested at different phases of the season. COD, change of direction.

Aspects of physical performance tested during seasonal phases are depicted in Figure 3. The most commonly reported test of acceleration was 10 m sprint time in the pre- (46%), in- (46%), and off-season (18%). The 5-0-5 test was the most common method of assessing agility/change of direction ability, reported by 55% of participants across all phases. Other agility/change of direction tests included the “*5-10-5*” and “*T-Test*.” Tests of anaerobic capacity included “*3s peak power Watt bike*,” “*anaerobic deficit 3x300s*,” “*ERU anaerobic running test*” and “*Watt bike 6s PPO*.” Participants most frequently used a 1.2 km time trial as an aerobic test during pre- (46%), in- (32%), and off-season (11%). The maximal Yo-Yo Intermittent Recovery Test was a commonly used alternative aerobic test in the pre- (16%) and in-season (16%).

Sums of 6–8 skinfolds were used during the pre-season (19%) and in-season (11%) to assess body composition. The Sit and Reach test was the only reported flexibility assessment. Muscular endurance tests included “*muscular capacity for calf*,” “*muscular capacity for hamstring*,” “*side bridge hold*,” “*inverted row*,” “*calf/hamstring/trunk/upper body pulling capacity*,” and “*sit ups (1 min)—max*.” Muscular power was assessed via a jump variation by 98% of participants during all phases. The counter-movement jump was the most commonly utilized jump variation during pre- (27%), in- (20%), and off-season (3%). Other jump variations used to test power included “*drop jump*,” “*broad jump*,” “*triple broad jump*,” “*Opto jump 15s RVJ*,” “*squat jump*,” “*RSI jumps*.” Non-jump power testing included “*VBT pull up, back squat/Bulgarian split squat and bench press*.” The bench press, squat and pull-up were the most common muscular strength tests in all phases. During the pre-season, these tests were used by 57, 51, and 43% of participants, respectively. Between 1 and 5 repetitions were used to measure strength on the reported lifts. Other strength tests included “*deadlift*,” “*power clean*,” “*prone row*,” “*bench pull*,” “*isometric mid-thigh pull*,” and “*maximal isometric hamstring bridge*.” Maximum velocity was tested by 40 m sprints with splits at 20 m or 30 m; or by an 80 m sprint with a radar gun. Other physical performance aspects that were tested include “*body mass*,” “*anthropometrics*,” “*fatigue monitoring*,” and “*maturation and motor control*.”

### Resistance Training

All participants believed resistance training was beneficial to female rugby performance. Participants indicated that players were obliged to resistance train in the pre-season (89%) and in-season (86%). During the off-season, 54% of participants stated that players had a choice whether to resistance train, and 41% stated players were obliged to train. Resistance training was prescribed 3 days a week in the pre-season (54%) and off-season (41%). In-season resistance training was prescribed 2 days per week by 68% of participants. Session duration typically lasted 45–60 min during the pre- (49%), in- (43%), and off-season (49%). Pre-season resistance training sessions were typically a combination of supervised and unsupervised (49%), or only supervised sessions (46%). Sessions were typically performed either pre-rugby training (49%) or on non-rugby training days (38%). In-season resistance training sessions were typically all supervised (54%) or a combination of supervised and unsupervised sessions (41%). These sessions were usually performed either pre-rugby training (51%) or on non-rugby training days (43%). Off-season resistance training was typically unsupervised (62%).

### Cardiovascular Training

Cardiovascular training was believed to be beneficial to female rugby performance (97%). Cardiovascular fitness training was mandatory during the pre- (78%) an in-season (70%). During the off-season phase, 54% of participants indicated that players had a choice whether to train cardiovascular fitness or not. Cardiovascular training was typically prescribed on 2 days a week in the pre- (49%), in- (49%), and off-season (51%). Pre- and in-season cardiovascular training sessions were generally a combination of supervised and unsupervised sessions, indicated by 59% of participants for both phases. Eighty-six percent of participants indicated that these sessions were integrated within rugby training sessions for both phases. Off-season cardiovascular training sessions were generally all unsupervised sessions (59%).

### Sprint Training

All participants stated that they believed sprint training was beneficial to female rugby performance. Sprint training was implemented by 95% of participants. Participants not implementing sprint training (5%) provided the following reasons:

“*not enough time […] to deliver speed component*”

and

“*more practical to complete conditioning within the gym due to my lack of availability as the S*&*C coach to complete pitch based sessions*.”

Responses for implementation of sprint training resulted in five main categories: (a) sprint performance enhancement, (b) injury risk reduction, (c) key rugby demand, (d) speed exposure, (e) miscellaneous (Table 3).

**Table 3:** Purpose of sprint training implementation.

**Main category**	**No. of responses**	**Select raw data representing responses to this question**
Sprint performance enhancement	14	Improve running efficiency, increase performance. Sprint training can improve anaerobic speed reserve. Technically develop running efficiency.
Injury risk reduction	10	Injury Prevention. Sprint training can potentially reduce injury risk. Injury prevention/resilience.
Key rugby demand	7	Maximal sprint is necessary in rugby demands. Sprint capacity is considered one of the key factors to be successful in rugby.
Speed exposure	7	Exposure to top speed. To expose athletes to maximal speed.
Miscellaneous	3	Build on stamina and fitness. Impove all aspects of the force-velocity curve. Sprint training is the simplest way to develop single limb strength.

Sprint training sessions were generally supervised during the pre- (70%) and in-season (68%). Pre-season sprint training prescription were typically one (38%) or two (48%) times per week. In-season sprint training prescription were one (54%) or two times (38%) per week. During the off-season, 41% of participants did not implement sprint training, while 30% implemented sessions once per week.

### Plyometric Training

All participants stated that they believed that plyometric training benefited female rugby performance. Plyometric training was implemented by 95% of participants. Participants who did not implement plyometric training (5%) provided the following reasons:

“*Our program is far from perfect, constantly growing and this will be layered in to what we do using an intentful prep pre-weights. Low-hanging fruit needed to be addressed first, speed and aerobic development*”

and

“*not enough time to deliver plyometric component*.”

Reasons for plyometric training implementation resulted in four main categories: (a) athletic performance enhancement, (b) injury risk reduction, (c) rugby performance enhancement, (d) miscellaneous (Table 4). During both pre- and in-season plyometric training sessions typically occurred as an integration within resistance training sessions (73, 70%), pre-sprint training (68, 70%), or pre-resistance training (57, 51%).

**Table 4:** Purpose of plyometric training implementation.

**Main category**	**No. of responses**	**Select raw data representing responses to this question**
Athletic performance enhancement	17	Improves acceleration, change of direction, jumping and sprinting performance. Develop stretch shorten cycle activity/muscle pre-excitation. To prevent energy leaks, enhance stiffness, neuromuscular efficiency, coordination.
Injury risk reduction	9	Injury prevention. Reduce injury. Minimizing injury risk.
Rugby performance enhancement	3	A key determinant of successful performance in many sporting actions in rugby.
Miscellaneous	2	Good for the core and creates muscles endurance. To develop the skill of movement.

### Recovery

Recovery sessions were believed to be beneficial for enhancing female rugby performance (89%) and were commonly implemented (76%). Participants who did not implement recovery sessions (24%) provided the following reasons:

“*Not full time with just that team, girls aren't full time or paid. Recovery is highly encouraged and information on how and what they should do is given and it is their choice if they do it (majority do)*”

and

“*Not enough time to implement only training twice a week”*

**Figure 4:**
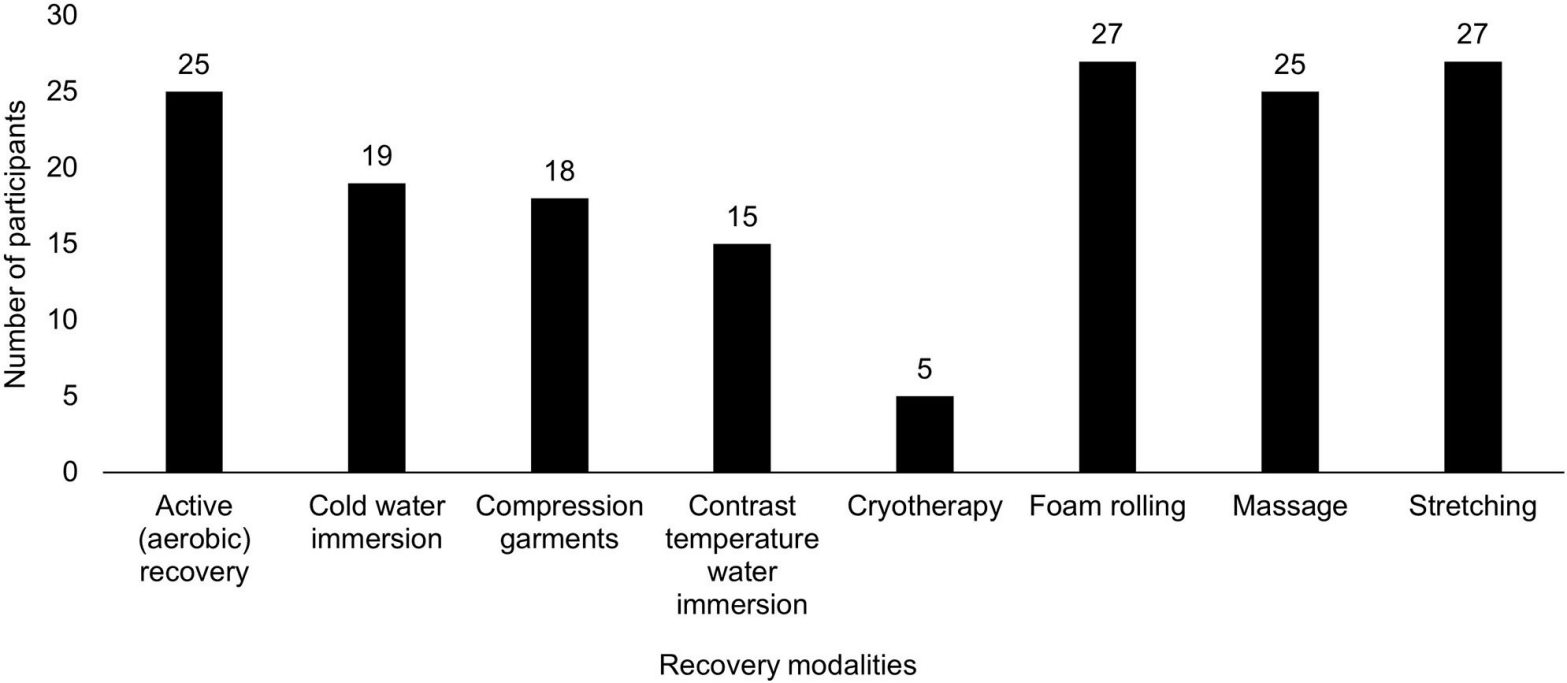
Recovery modalities.

Recovery modalities are described in Figure 4. Reasons for implementing recovery sessions resulted in six main categories: (a) improve recovery time, (b) physiological regeneration, (c) injury risk reduction, (d) psychological regeneration, (e) performance enhancement, and (d) miscellaneous (Table 5). Implementation of recovery sessions into the weekly micro-cycle were performed on non-training days (62%), pre- (24%), and post- (30%) resistance training, pre- (16%) and post- (41%) rugby training and post-match play (59%).

**Table 5:** Purpose of recovery session implementation.

**Main category**	**No. of responses**	**Select raw data representing responses to this question**
Improve recovery time	8	Improve the ability of the athlete to recover between sessions. Because the faster the athletes recover, the sooner they are able to train again. Lesson the time it takes players to recover.
Physiological regeneration	6	Promote tissue regeneration, nervous system homeostasis. Decrease inflammation. Flush lymphatic system.
Injury risk reduction	6	To prevent over-training and injuries. Reduce injury risk. Reduce the risk of injury
Psychological regeneration	2	To improve mental perception of recovery. Well-being.
Performance enhancement	2	Improve performance. Ensure optimum performance.
Miscellaneous	1	Improve their longevity in the sport.

### Monitoring and Sport Science Technology

**Figure 5:**
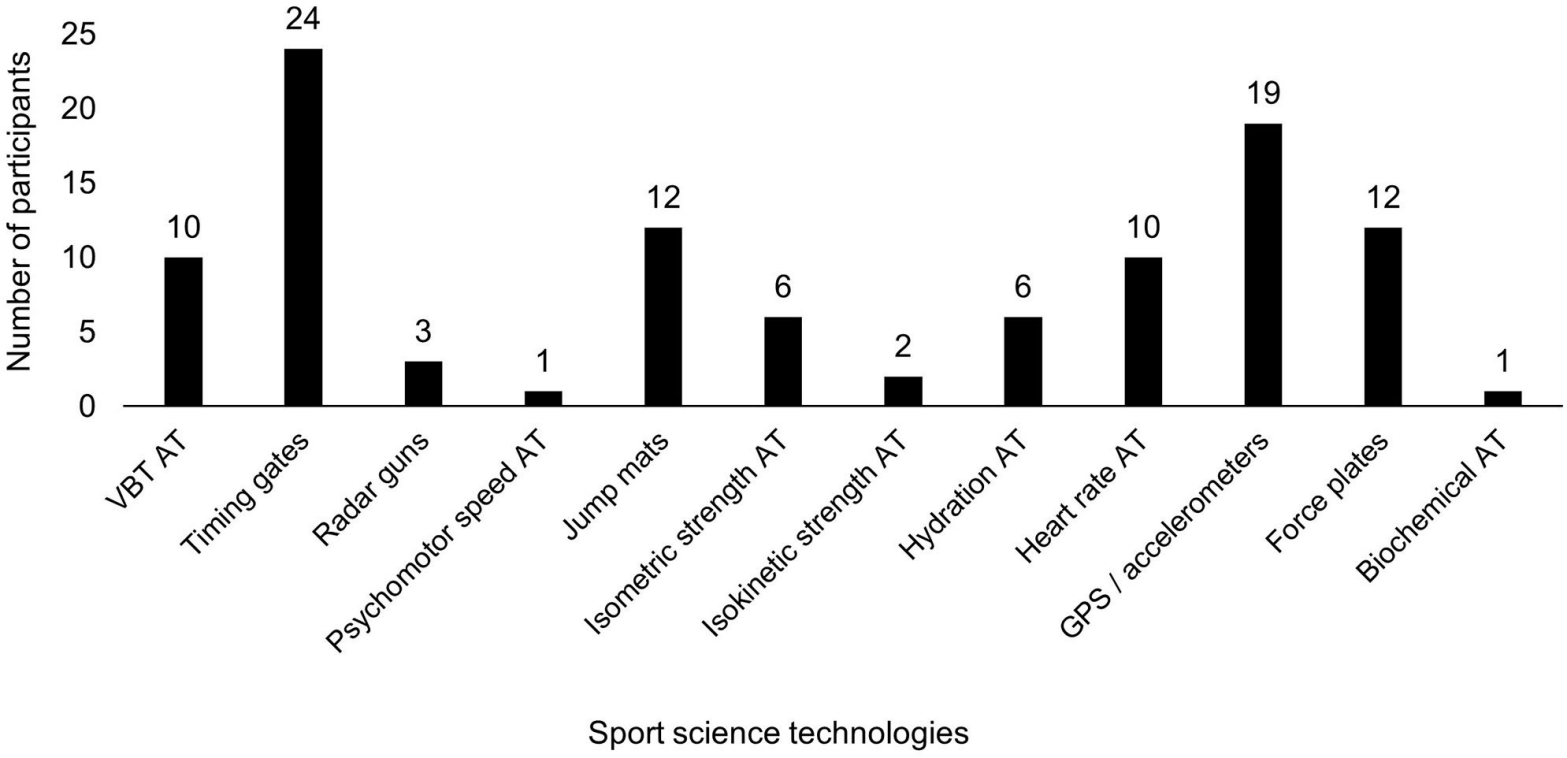
Sport science technologies utilized by participants. AT, assessment tools; GPS, global positioning systems; VBT, velocity-based training.

Monitoring athlete wellness (e.g., mood, stress, muscle soreness, and sleep) was believed to be beneficial for assessing recovery status in rugby (92%) and undertaken by 76% of participants. Wellness monitoring frequency occurred every day (24%), every session (22%), every week (19%), multiple days per week (14%), or was not monitored (22%). Monitoring of menstrual cycle phase was performed by 22% of participants. Training and match loads were monitored by 76% of participants. Sport science technologies to assist decision making are described in Figure 5. Responses of how sport science technologies assist participants resulted in three main categories: (a) informs practice, (b) monitoring, (c) miscellaneous (Table 6).

**Table 6:** Purpose of sport science technologies.

**Main category**	**No. of responses**	**Select raw data representing responses to this question**
Informs practice	20	Supplies me and the sports coaches with objective data to inform session planning. To provide data to help objectify our coaching decisions. Plan and prescribe for future training sessions.
Monitoring	15	To monitor the athlete well-being and readiness to train and play across the weeks. Load monitoring. Track improvements in performance.
Miscellaneous	6	Provides much more accurate results compared to measuring manually. !!break Providing tools to give them [athletes] an advantage gets me buy in. To support discussions with athletes. They make it easier to collect simple data.

### Unique Aspects of Consideration Within Female Rugby

Responses of unique aspects of consideration (e.g., contextual, physiological, and physical) in the physical preparation of female rugby resulted in eight main categories: (a) psycho-social aspects, (b) menstrual cycle, (c) physical differences, (d) external commitments, (e) variability, (f) education, (g) limited access and, (h) miscellaneous (Table 7).

**Table 7:** Unique aspects of consideration in female rugby.

**Main category**	**No. of responses**	**Select raw data representing responses to this question**
Psycho-social aspects	15	More social aspects need to be integrated. Psychologically it is very important to pay attention to players within women's rugby, and to have regular conversation to see how they are feeling/progressing. They wear their emotions on their sleeve more than men do and therefore may require an individual conversation more often. The stigmas associated with lifting weights within a female population.
Menstrual cycle	8	I think it is important to be aware of how it can influence their mood, energy levels, and physical performance and therefore adjust expectations of the athlete at certain times in their cycle.
Physical differences	8	Gym based training experience [is often low]. Their strength parameters and benchmarks are lower than men's. Women tend to have a more accentuated/frequent knee valgus and this might be a factor of [injury] risk.
External commitments	4	Children and juggling full time work!!! Ladies are amazing in that they run their household, work, and train. Even our contracted International level players have full-time work or study commitments in-season.
Variability	4	Huge variance in … attitude within S&C, attendance, knowledge. The girls selected are of a vast range of skill and athleticism as well which makes it difficult to program.
Education	3	Education and understanding as to why physical preparation is so important for athletes. Coaching staff are still quite old school meaning most of what you want to achieve as an S&C coach is hindered because of lack of [sports coach] education.
Limited access	2	No[t] having access to players on a regular basis.
Miscellaneous	2	Because it is underfunded, athletes are not as professional as you want them to be which makes it a challenge for them to do anything outside of training.

## Discussion

The present study is the first to comprehensively describe the physical preparation practices in female rugby. A total of 37 participants responded to the questionnaire, this is in-line with previous studies investigating male athletes which have received between 20 and 43 responses (Ebben and Blackard, 2001; Simenz et al., 2005; Gee et al., 2011; Jones T. et al., 2016, 2017; Robinson et al., 2019). This study accounted for a multitude of aspects that influence physical performance (i.e., seasonal changes in physical preparation, recovery, monitoring and sport science technology, and unique considerations in female rugby). The most common physical performance tests included acceleration, aerobic capacity, muscular strength, and maximum velocity measures. Testing was performed most often in pre- and in-season phases. Resistance, cardiovascular, sprint and plyometric training, and recovery sessions were believed to be important to enhancing female rugby performance and implemented by most participants. Practitioners typically reported the purpose of sport science technologies were to inform current practice (54%) (e.g., provide data to plan future sessions) or monitor players (41%) (e.g., tracking training load and well-being). Menstrual cycle phase was not commonly monitored, with only 22% of participants tracking it. The most frequently reported unique considerations in female rugby codes included psycho-social aspects (41%), the menstrual cycle (22%), and physical differences (22%). To the authors knowledge, this is the first study to provide an overview of physical preparation practices exclusively in female athletes.

The importance of physical testing appeared to vary during different seasonal phases. The most commonly assessed aspects of physical fitness in the pre-season were aerobic capacity and muscular strength, while in-season, acceleration and muscular strength took precedence. These findings reflect practices in elite male rugby, where despite testing not being sub-categorized into seasonal phases, aspects of physical fitness were assessed by >78% of participants (Jones T. et al., 2016). Differences in testing emphasis in female rugby from pre-season to in-season may reflect training emphasis changes across phases which is in-line with other female team sports (Brown and Lopez, 2016; Duggan et al., 2020). Overall, physical testing was predominately conducted in the pre- (97%) and in-season (86%) phases, with only 23% of participants fitness testing in the off-season. Reasons for off-season testing may be to assess and mitigate negative changes in physiological characteristics (Stokes et al., 2020). This is similar to elite male rugby, where percentages of participants conducting physical testing dropped from 95% during the in-season to 53% in the off-season (Jones T. et al., 2016). Both male and female rugby may face similar logistical issues in the off-season with a lack of player access.

Strength and resistance training are considered beneficial to female rugby performance (Reilly, 1997; Argus et al., 2012). Up to 81% of participants indicated that 45–60 min resistance training sessions were prescribed 2–3 days a week across all season phases (81%). This is similar to findings in male academy rugby league where U16 players were exposed to ~3 resistance training sessions per week of ~50 min in both pre- and in-season phases (McCormack et al., 2020). Conversely, U19 rugby league players were exposed to greater resistance training frequency during the pre-season (4.4 ± 0.7) compared to the middle of the in-season (3.2 ± 0.6) (McCormack et al., 2020) which may highlight that greater sport science provision to male players is afforded as they progress through the pathway. Poor access to players was a commonly reported logistical challenge for practitioners working within female rugby (Tables 2, 7). Available contact time should therefore emphasize movements that yield the best return on investment. Consistency in exposure to, and development of, the athletic motor skill competencies (e.g., lower body uni- and bi-lateral concentric and eccentric training, upper body pushing and pulling in both vertical and horizontal planes) will act as the foundation for more complex future training (e.g., weightlifting and high velocity movements) (Lloyd et al., 2015). Additionally, implementation of injury risk reduction strategies specific to the female rugby player should be embedded within a comprehensive physical preparation programme. For example, as anterior cruciate ligament injuries are a burden in female rugby (Fuller et al., 2017; Toohey et al., 2019), incorporation of neuromuscular training (e.g., landing stabilization) should be prioritized (Petushek et al., 2019).

Despite aerobic capacity underling the ability for repeated high intensity efforts, which are key demands of rugby, the requirements of high aerobic capacity in both male and female rugby players are uncertain (Duthie et al., 2003). Participants may use aerobic capacity testing in the pre-season in order to prescribe cardiovascular fitness training and inform session parameters (e.g., work interval intensity and duration, rest interval intensity and duration, number of sets and reps) (Buchheit and Laursen, 2013). This may be used to create underlying physiological cardiovascular adaptations in order to ensure that aerobic capacity is not a limiting factor to performance (Glaister, 2005). During the in-season, cardiovascular fitness sessions were integrated within rugby training (86%), this time-efficient design allows technical and tactical skills to be developed in unison with positive physiological adaptation. In a mixed-sex sample of adolescent handball players, implementation of small-sided games (2 sessions per week of 2–4 games lasting 2 min 30 s−4 min duration interspersed with 30 s passive recovery over 10 weeks) have been used to create improvements in both repeat sprint ability and 30–15 Intermittent Fitness Test scores by ~4 and ~6%, respectively (Buchheit et al., 2009). Strong working relationships between rugby coaches and practitioners are vital to facilitate these types of collaborations which enhance technical, tactical, and physiological adaptations.

Rugby is physiologically and biomechanically demanding, resulting in muscle damage and inflammation (Tavares et al., 2017). These stress responses can negatively influence perceptual and neuromuscular function, which can persist for up to 4 days (Tavares et al., 2017). Furthermore, recent research suggests that female sex hormones influence the physiological recovery response (Hackney et al., 2019). Previous research has demonstrated the effectiveness of recovery modalities (e.g., cold-water immersion and compression garments) for enhancing creatine kinase clearance and neuromuscular function, decreasing delayed-onset muscle soreness and improving perceived recovery (Tavares et al., 2017). The discrepancy between participants who perceived recovery sessions as beneficial to rugby performance (89%) and those who implemented sessions (76%) may be due to contextual factors (e.g., developing nature of female rugby, limited time and resources), thus player education on at-home recovery session modalities and their importance may be a practical solution to an applied problem.

Wellness monitoring was deemed important to female rugby performance by 92% of participants. Although tracking of wellness, match and training loads were found to be prevalent (76%), which aligns with a recent training load survey in amateur male and female rugby union (Griffin et al., 2020), menstrual cycle phase was only monitored by 22% of participants. There is an emerging body of evidence suggesting that the menstrual cycle may influence athletic performance (Findlay et al., 2020; McNulty et al., 2020), internal training load (Cristina-Souza et al., 2019) and injury risk (Herzberg et al., 2017). This evidence, coupled with participant views that the menstrual cycle is an important aspect of consideration in female rugby (Table 7), suggests that monitoring of cycle phase, associated symptoms, training load, and wellness may be crucial for sex-specific sports performance. Previous literature has suggested a laboratory-based three-step procedure for menstrual cycle phase verification (Schaumberg et al., 2017). As this procedure may not be feasible in applied settings, an individualized approach to monitoring menstrual cycle-related symptoms may be more appropriate (McNulty et al., 2020). Future longitudinal research investigating the relationships between the menstrual cycle, training loads and wellness may be a key unique feature for developing understanding of performance factors in female sport.

Psycho-social aspects were a key unique feature of female rugby considerations. To the authors' knowledge, there are no sex-specific coaching effectiveness literature pertaining to psycho-social aspects (Sargent and Barker, 2018). Despite this, psycho-social sex differences have been described in the literature, suggesting that empathy, and connections are of higher value to females compared to males (Cunningham and Roberts, 2012). This literature is echoed by participants who have stated that players require more individual contact to support emotional well-being (Table 7). Considering this, practitioners should be cognizant of coaching style (e.g., autonomy-supportive vs. controlling) (Amorose and Anderson-Butcher, 2015) and should emphasize building strong rapport and professional relationships with female players. Additionally, a key discussion point identified within female rugby is the gender identity paradox where players may display feminine behaviors while engaged in a physically demanding sport that has been traditionally aligned with masculinity (Joncheray et al., 2016). Practitioners should consider how this paradox could affect players willingness to engage in physical preparation programmes due to female athletes' desire to achieve both high performance levels and simultaneously conform to identified feminine behaviors and body type. Practitioners should therefore be aware of these factors when emphasizing the importance of adherence to physical preparation programmes to female players.

Furthermore, physical differences were stated to be a unique consideration by 22% of participants. Some participant responses highlighted low (and variable) training age, low strength, and accentuated knee valgus of female rugby players when compared to men. Reduction of excessive knee valgus may therefore be an important consideration for injury risk reduction in female rugby. As greater lower body strength may decrease knee valgus during both jumping (Jacobs and Mattacola, 2005) and squatting (Claiborne et al., 2006) tasks, lower body strength development is recommended for the female rugby player. Appropriate coaching and training interventions for the female athlete may eliminate any relative strength deficit compared to males (Burger and Burger, 2002). Therefore, early introduction of periodised strength training is vital to the safe athletic development of the female rugby player.

The duration of season phases varied greatly. Due to limitations in questionnaire design, pre- and in-season phase length data were not all able to be captured to the specific week. The large variation seems to be context-specific, as phase duration of female rugby varies across countries and codes. For example, the Australian 2019 Super W rugby union season was played over 5 rounds compared to the 18-round 2018–19 Premier 15 rugby union season in England. This contrasts with the 7-round 2019 Men's National Rugby Championship (Australia) and 22-round 2018–19 Men's Premiership Rugby season (England). The differences may highlight the current landscape of female sport, the developing professionalization, infrastructure and participation in female rugby in various countries. Therefore, practitioners should consider their sporting setting when applying presented findings into practice.

Results from this study have highlighted great heterogeneity in physical preparation practices in female rugby which may signify context-specific constraints or a lack of consensus on a best-practice approach which is similar to findings investigating male athletes (Ebben and Blackard, 2001; Ebben et al., 2004, 2005; Simenz et al., 2005; Gee et al., 2011; Winwood et al., 2011; Jones T. et al., 2016; Pote and Christie, 2016; Crowley et al., 2018; Robinson et al., 2019). Some participants of this study may work within large multidisciplinary teams where responsibilities of certain aspects of physical preparation may fall outside of their remit, in these cases data may be skewed and should therefore be viewed with caution. As rugby codes exhibit differences with respect to physical demands, the practical application of these research findings should be implemented with consideration to the practitioners' context. Due to recruitment methods, the included participants may not constitute a representative sample, discretion should therefore be applied when interpreting results. As 92% of participants surveyed worked with international, national or regional/state level players, practitioners now have a source of data describing physical preparation practices in a high standard of female rugby. Future researchers could use the presented data to design experimental protocols examining the effect of physical preparation practices on various aspects of performance or investigate unique considerations of female rugby in further detail, and their relationships with performance.

## Practical Applications

Physical preparation practitioners working with female rugby codes, or games with similar demands, can use presented findings as a resource to further inform and develop current practices. As practitioners in female sport often have limited player contact time, due to low financial investment and other factors, emphasis on time efficient training must be stressed. Player education on the physical preparation process may assist in compliance with training when supervision is not feasible. Strong professional relationships with players and coaching staff may assist in the integration of technical, tactical and physical preparation elements. Menstrual cycle phase and associated symptom tracking should be integrated within wellness monitoring, as a consideration for the female athlete. Furthermore, practitioners should consider the unique aspects of female rugby, such as psycho-social aspects and physical differences.

## Data Availability Statement

The raw data supporting the conclusions of this article will be made available by the authors, without undue reservation.

## Ethics Statement

This study involving human participants was reviewed and approved by Leeds Beckett University Research Ethics Committee. The participants provided their written informed consent to participate in this study.

## Author Contributions

OH, SE, GR, and BJ: conceptualization and design. OH and BN: data interpretation and analysis. OH: original draft preparation. OH, BN, SE, GR, and BJ: reviewing and editing. All authors contributed to the the article and approved the submitted version.

## Conflict of Interest

The authors declare that the research was conducted in the absence of any commercial or financial relationships that could be construed as a potential conflict of interest.
