# Supplementary material for: Physical Preparation in Female Rugby Codes: An Investigation of Current Practices
Source: Front Sports Act Living. 2020 Nov 26;2:584194. doi: 10.3389/fspor.2020.584194 (PMC7739696; doi:10.3389/fspor.2020.584194)
Supplement: Supplementary file 1 [file Data_Sheet_1.pdf]

Data Sheet 1: Copy of Questionnaire

Physical Preparation Practices in Women's Rugby Codes

INFORMATION AND CONSENT

Invitation to take part
You are being invited to participate in a women's rugby code (rugby union, rugby league, rugby sevens) research project. Leeds Beckett University are investigating the physical preparation practices of women's rugby codes at different levels of competition.

This study is part of a PhD project which is partly funded by the Rugby Football Union. You will not be required to divulge any personally identifying information. The collected data will be used for research purposes only, therefore none of the collected data will be directly disclosed with the Rugby Football Union.

Before you decide whether to take part it is important for you to understand the purpose of the research and what will be involved. Please take time to read the following information carefully. This study follows the ethical guidelines of Leeds Beckett University and has received ethical approval from the Local Research Ethics Coordinator, in line with the University's Research Ethics Policy.

What is the purpose of the project?
Women's rugby codes are rapidly growing in popularity, participation and professionalism. Despite this little is known regarding the physical preparation practices of women's rugby code athletes. If players do not receive adequate physical preparation support, then sporting performance may be negatively affected and injury risk may be increased. Therefore, the purpose of this project is to understand the physical preparation practices of different women's rugby codes.

Why have you been invited to take part?
You have been invited to complete this survey because you are a currently the lead physical preparation practitioner (e.g. strength and conditioning coach, athletic development coach, physical performance coach) responsible for delivery to women's rugby athletes.

What will happen if I take part?
You will be asked to complete an electronic survey on your computer or mobile device which takes approximately 15 minutes to answer. The survey asks questions about testing, training, recovery and

monitoring practices specific to the female rugby athlete's you work with.

What are the benefits of taking part?
You will be providing information which may assist in the development of physical preparation needs in women's rugby codes to enhance performance.

What are the risks of taking part?
There is minimal risk in taking part. All participants will remain anonymous as data will not be collected with any identifying information.

What if I am not happy with how the research was conducted?
If there is anything you are unhappy with while taking part in the project, you should report it to the principle investigator or director of studies.

Do I have to take part?
No participation is completely voluntary.

Once I take part, can I change my mind?
You are still free to withdraw at any time, for any reason and you will not be asked to explain your reasons for doing so. In order to withdraw your participation, simply contact the principle investigator on the email address below.

Will my taking part in this project be kept confidential?
Yes. Participants will not be identifiable in any way. Participants will not be required to divulge any personally identifying information.

What will happen to the results of the research project?
The information from this study will be shared anonymously with the wider sport science community through conference presentation and peer-review publication.

Contact details
Principal investigator
Omar Heyward
O.Heyward@leedsbeckett.ac.uk

Director of Studies
Professor Ben Jones
B.jones@leedsbeckett.ac.uk

Local Research Ethics Coordinator
Dr. Jason Tee
J.C.Tee@leedsbeckett.ac.uk

- ☐ I have read and understand the participant information sheet
- ☐ I have had sufficient time to consider whether or not to participate in the study
- ☐ I understand that taking part in this study is voluntary and I may withdraw from the study at any time
- ☐ I consent to the researchers collecting and processing information about the physical preparation practices that I prescribe
- ☐ I understand that there is a small risk of data breach associated with this research as it is conducted online
- ☐ I understand that researchers will do all they can to ensure that my participation in this study is confidential and that no personal identifying material will be used in any reports
- ☐ I consent to the researchers publishing anonymous data in peer-reviewed journals and conference presentations
- ☐ I know who to contact if I have any questions or concerns about the study

<u>BACKGROUND DETAILS</u>

1. How old are you?

____

2. What is your sex?
   - ☐ Male
   - ☐ Female
   - ☐ Prefer not to disclose

3. What is the title of your current physical preparation role (e.g. strength and conditioning coach, athletic development coach, performance specialist)?

____

4. In which country do you currently work?

____

5. What is the highest level of education you have completed?
   - ☐ High School or equivalent
   - ☐ Bachelor's Degree
   - ☐ Master's Degree
   - ☐ Doctoral Degree

6. What is the area of specialisation in your highest completed education? (e.g. sport / exercise science, human movement science, high performance)

____

7. Have you achieved any physical preparation (e.g. UKSCA, ASCA, NSCA) certifications?

- ☐ Yes
- ☐ No

8. What certifications have you achieved?

____

9. Which women's rugby codes do you currently work with? (Select all that apply)

- ☐ Rugby Union
- ☐ Rugby League
- ☐ Rugby Sevens

10. Do you work with teams or individual athletes?

- ☐ Team
- ☐ Individual
- ☐ Both Team and Individual

11. What level of competition do the athletes you work with compete at? (If working with athletes of multiples levels, please select the predominant level)

- ☐ International
- ☐ National
- ☐ Regional / State
- ☐ Recreational / Local

<u>PRE-SEASON</u>

12. How many weeks in duration is the pre-season phase?

____

13. Please check relevant boxes to outline a typical pre-season training week.

Match Play	☐ Monday	☐ Tuesday	☐ Wednesday	☐ Thursday	☐ Friday	☐ Saturday	☐ Sunday
Rugby Training	☐ Monday	☐ Tuesday	☐ Wednesday	☐ Thursday	☐ Friday	☐ Saturday	☐ Sunday
Resistance Training	☐ Monday	☐ Tuesday	☐ Wednesday	☐ Thursday	☐ Friday	☐ Saturday	☐ Sunday
Cardiovascular Training	☐ Monday	☐ Tuesday	☐ Wednesday	☐ Thursday	☐ Friday	☐ Saturday	☐ Sunday
Speed Development	☐ Monday	☐ Tuesday	☐ Wednesday	☐ Thursday	☐ Friday	☐ Saturday	☐ Sunday
Plyometric Training	☐ Monday	☐ Tuesday	☐ Wednesday	☐ Thursday	☐ Friday	☐ Saturday	☐ Sunday

14. Do you conduct any pre-season physical performance testing of your women's rugby athletes?

- ☐ Yes
- ☐ No

15. If you selected ‘No’ to question 14, please describe why physical performance tests are not performed.

____

16. What aspects of physical performance are tested?

- ☐ Acceleration
- ☐ Agility
- ☐ Anaerobic capacity
- ☐ Aerobic capacity
- ☐ Body composition
- ☐ Change of direction
- ☐ Flexibility
- ☐ Muscular endurance
- ☐ Muscular strength
- ☐ Muscular power
- ☐ Maximum speed
- ☐ Other

17. If you selected ‘Other’ to question 16, describe the other physical performance aspects that are tested.

____

18. What are the physical tests used to assess the physical aspects listed in question 16 (and/or question 17)?

____

19. Do you believe resistance training (e.g. free weights, machine weights, weighted implements) benefits rugby performance?

- ☐ Yes
- ☐ Not sure
- ☐ No

20. Are your athletes obliged to resistance train in the pre-season phase?

- ☐ Yes
- ☐ No
- ☐ Athlete's choice

21. During the pre-season phase, how many resistance training sessions do you typically prescribe per week?

- ☐ 0 sessions per week
- ☐ 1 session per week
- ☐ 2 sessions per week
- ☐ 3 sessions per week
- ☐ 4 sessions per week
- ☐ ≥ 5 sessions per week

22. During the pre-season phase, what is the supervision level of resistance training sessions?

- ☐ Supervised sessions
- ☐ Unsupervised sessions
- ☐ Combination of supervised and unsupervised sessions

23. During the pre-season phase, what is the typical duration of resistance training sessions?

- ☐ 0-30 minutes
- ☐ 30-45 minutes
- ☐ 45-60 minutes
- ☐ 60-75 minutes
- ☐ > 75 minutes

24. During the pre-season phase, where do resistance training sessions fit into a typical training schedule?

- ☐ Non rugby training days
- ☐ Stand alone sessions (i.e. > 2 hours rest between same-day training sessions)
- ☐ Pre-rugby training sessions (i.e. < 2 hours rest before rugby session)
- ☐ Post-rugby training sessions (i.e. < 2 hours rest after rugby session)

25. Do you believe cardiovascular training benefits rugby performance?

- ☐ Yes
- ☐ Not sure
- ☐ No

26. Are your athletes obliged to undertake training to develop cardiovascular fitness in the pre-season phase?

- ☐ Yes
- ☐ No
- ☐ Athlete's choice

27. During the pre-season phase, how many cardiovascular training sessions do you typically prescribe per week?

- ☐ 0 sessions per week
- ☐ 1 session per week
- ☐ 2 sessions per week
- ☐ 3 sessions per week
- ☐ 4 sessions per week
- ☐ ≥ 5 sessions per week

28. During the pre-season phase, what is the supervision level of cardiovascular training sessions?

- ☐ Supervised sessions
- ☐ Unsupervised sessions
- ☐ Combination of supervised and unsupervised sessions

29. During the pre-season phase, where do cardiovascular sessions fit into a typical training schedule?

- ☐ Stand alone sessions (i.e. > 2 hours rest between same-day training sessions)
- ☐ Pre-rugby training sessions (i.e. < 2 hours rest before rugby session)
- ☐ Post-rugby training sessions (i.e. < 2 hours rest after rugby session)
- ☐ Integrated within rugby training session (i.e. short blocks between rugby elements)

30. Do you believe that sprint training benefits rugby performance?

- ☐ Yes
- ☐ Not sure
- ☐ No

31. Please describe why sprint training is/ is not implemented into your programme.

____

32. How many sessions per week is sprint training typically implemented into your pre-season?

- ☐ 0 sessions per week
- ☐ 1 session per week
- ☐ 2 sessions per week
- ☐ 3 sessions per week
- ☐ 4 sessions per week
- ☐ ≥ 5 sessions per week

33. During the pre-season phase, what is the supervision level of sprint training sessions?

- ☐ Supervised sessions
- ☐ Unsupervised sessions
- ☐ Combination of supervised and unsupervised sessions

34. Do you believe that plyometric training benefits rugby performance?

- ☐ Yes
- ☐ Not sure
- ☐ No

35. Do you implement plyometric training into your programme?

- ☐ Yes
- ☐ No

36. Please describe why plyometric training is/is not implemented into your programme.

____

37. During the pre-season phase, where do plyometric training sessions fit into a typical training schedule?

- ☐ Stand alone sessions (i.e. > 2 hours rest between same-day training sessions)
- ☐ Pre-rugby training sessions (e.g. in warms ups)
- ☐ Integrated within rugby training session (i.e. short blocks between rugby elements)
- ☐ Pre-resistance training sessions (e.g. in warm ups)
- ☐ Integrated within resistance training sessions (e.g. contrast / complex methods)
- ☐ Pre-sprint training sessions (e.g. in warm ups)
- ☐ Integrated within sprint training sessions (i.e. short blocks between sprint training elements)

<u>IN-SEASON</u>

38. How many weeks in duration is the in-season phase?

____

39. Please check relevant boxes to outline a typical in-season training week.

Match Play	☐ Monday	☐ Tuesday	☐ Wednesday	☐ Thursday	☐ Friday	☐ Saturday	☐ Sunday
Rugby Training	☐ Monday	☐ Tuesday	☐ Wednesday	☐ Thursday	☐ Friday	☐ Saturday	☐ Sunday
Resistance Training	☐ Monday	☐ Tuesday	☐ Wednesday	☐ Thursday	☐ Friday	☐ Saturday	☐ Sunday
Cardiovascular Training	☐ Monday	☐ Tuesday	☐ Wednesday	☐ Thursday	☐ Friday	☐ Saturday	☐ Sunday
Speed Development	☐ Monday	☐ Tuesday	☐ Wednesday	☐ Thursday	☐ Friday	☐ Saturday	☐ Sunday
Plyometric Training	☐ Monday	☐ Tuesday	☐ Wednesday	☐ Thursday	☐ Friday	☐ Saturday	☐ Sunday

40. Do you conduct any in-season physical performance testing of your women's rugby athletes?

- ☐ Yes
- ☐ No

41. If you selected ‘No’ to question 40, please describe why physical performance tests are not performed.

____

42. What aspects of physical performance are tested?

- ☐ Acceleration
- ☐ Agility
- ☐ Anaerobic capacity
- ☐ Aerobic capacity
- ☐ Body composition
- ☐ Change of direction
- ☐ Flexibility
- ☐ Muscular endurance
- ☐ Muscular strength
- ☐ Muscular power
- ☐ Maximum speed
- ☐ Other

43. If you selected ‘Other’ to question 42, describe the other physical performance aspects that are tested.

____

44. What are the physical tests used to assess the physical aspects listed in question 42 (and/or question 43)?

____

45. Are your athletes obliged to resistance train in the pre-season phase?

- ☐ Yes
- ☐ No
- ☐ Athlete's choice

46. During the in-season phase, how many resistance training sessions do you typically prescribe per week?

- ☐ 0 sessions per week
- ☐ 1 session per week
- ☐ 2 sessions per week
- ☐ 3 sessions per week
- ☐ 4 sessions per week
- ☐ ≥ 5 sessions per week

47. During the in-season phase, what is the supervision level of resistance training sessions?

- ☐ Supervised sessions
- ☐ Unsupervised sessions
- ☐ Combination of supervised and unsupervised sessions

48. During the in-season phase, what is the typical duration of resistance training sessions?

- ☐ 0-30 minutes
- ☐ 30-45 minutes
- ☐ 45-60 minutes
- ☐ 60-75 minutes
- ☐ > 75 minutes

49. During the in-season phase, where do resistance training sessions fit into a typical training schedule?

- ☐ Non rugby training days
- ☐ Stand alone sessions (i.e. > 2 hours rest between same-day training sessions)
- ☐ Pre-rugby training sessions (i.e. < 2 hours rest before rugby session)
- ☐ Post-rugby training sessions (i.e. < 2 hours rest after rugby session)

50. Are your athletes obliged to undertake training to develop cardiovascular fitness in the in-season phase?

- ☐ Yes
- ☐ No
- ☐ Athlete's choice

51. During the in-season phase, how many cardiovascular training sessions do you typically prescribe per week?

- ☐ 0 sessions per week
- ☐ 1 session per week
- ☐ 2 sessions per week

- ☐ 3 sessions per week
- ☐ 4 sessions per week
- ☐ ≥ 5 sessions per week

52. During the in-season phase, what is the supervision level of cardiovascular training sessions?
    - ☐ Supervised sessions
    - ☐ Unsupervised sessions
    - ☐ Combination of supervised and unsupervised sessions

53. During the in-season phase, where do cardiovascular sessions fit into a typical training schedule?
    - ☐ Stand alone sessions (i.e. > 2 hours rest between same-day training sessions)
    - ☐ Pre-rugby training sessions (i.e. < 2 hours rest before rugby session)
    - ☐ Post-rugby training sessions (i.e. < 2 hours rest after rugby session)
    - ☐ Integrated within rugby training session (i.e. short blocks between rugby elements)

54. How many sessions per week is sprint training typically implemented into your in-season?
    - ☐ 0 sessions per week
    - ☐ 1 session per week
    - ☐ 2 sessions per week
    - ☐ 3 sessions per week
    - ☐ 4 sessions per week
    - ☐ ≥ 5 sessions per week

55. During the in-season phase, what is the supervision level of sprint training sessions?
    - ☐ Supervised sessions
    - ☐ Unsupervised sessions
    - ☐ Combination of supervised and unsupervised sessions

56. During the in-season phase, where do plyometric training sessions fit into a typical training schedule?
    - ☐ Stand alone sessions (i.e. > 2 hours rest between same-day training sessions)
    - ☐ Pre-rugby training sessions (e.g. in warms ups)
    - ☐ Integrated within rugby training session (i.e. short blocks between rugby elements)
    - ☐ Pre-resistance training sessions (e.g. in warm ups)
    - ☐ Integrated within resistance training sessions (e.g. contrast / complex methods)
    - ☐ Pre-sprint training sessions (e.g. in warm ups)
    - ☐ Integrated within sprint training sessions (i.e. short blocks between sprint training elements)

OFF-SEASON

57. How many weeks in duration is the off-season phase?

____

58. Please check relevant boxes to outline a typical off-season training week.

Match Play	☐ Monday	☐ Tuesday	☐ Wednesday	☐ Thursday	☐ Friday	☐ Saturday	☐ Sunday
Rugby Training	☐ Monday	☐ Tuesday	☐ Wednesday	☐ Thursday	☐ Friday	☐ Saturday	☐ Sunday
Resistance Training	☐ Monday	☐ Tuesday	☐ Wednesday	☐ Thursday	☐ Friday	☐ Saturday	☐ Sunday
Cardiovascular Training	☐ Monday	☐ Tuesday	☐ Wednesday	☐ Thursday	☐ Friday	☐ Saturday	☐ Sunday
Speed Development	☐ Monday	☐ Tuesday	☐ Wednesday	☐ Thursday	☐ Friday	☐ Saturday	☐ Sunday
Plyometric Training	☐ Monday	☐ Tuesday	☐ Wednesday	☐ Thursday	☐ Friday	☐ Saturday	☐ Sunday

59. Do you conduct any off-season physical performance testing of your women's rugby athletes?

- ☐ Yes
- ☐ No

60. If you selected 'No' to question 59, please describe why physical performance tests are not performed.

____

61. What aspects of physical performance are tested?

- ☐ Acceleration
- ☐ Agility
- ☐ Anaerobic capacity
- ☐ Aerobic capacity
- ☐ Body composition
- ☐ Change of direction
- ☐ Flexibility
- ☐ Muscular endurance
- ☐ Muscular strength
- ☐ Muscular power
- ☐ Maximum speed
- ☐ Other

62. If you selected 'Other' to question 61, describe the other physical performance aspects that are tested.

____

63. What are the physical tests used to assess the physical aspects listed in question 61 (and/or question 62)?

____

64. Are your athletes obliged to resistance train in the off-season phase?

- Yes
- No
- Athlete's choice

65. During the off-season phase, how many resistance training sessions do you typically prescribe per week?

- 0 sessions per week
- 1 session per week
- 2 sessions per week
- 3 sessions per week
- 4 sessions per week
- ≥ 5 sessions per week

66. During the off-season phase, what is the supervision level of resistance training sessions?

- Supervised sessions
- Unsupervised sessions
- Combination of supervised and unsupervised sessions

67. During the off-season phase, what is the typical duration of resistance training sessions?

- 0-30 minutes
- 30-45 minutes
- 45-60 minutes
- 60-75 minutes
- > 75 minutes

68. Are your athletes obliged to undertake training to develop cardiovascular fitness in the off-season phase?

- Yes
- No
- Athlete's choice

69. During the off-season phase, how many cardiovascular training sessions do you typically prescribe per week?

- 0 sessions per week
- 1 session per week
- 2 sessions per week
- 3 sessions per week
- 4 sessions per week
- ≥ 5 sessions per week

70. During the off-season phase, what is the supervision level of cardiovascular training sessions?

- Supervised sessions
- Unsupervised sessions

- ☐ Combination of supervised and unsupervised sessions

71. How many sessions per week is sprint training typically implemented into your off-season?
- ☐ 0 sessions per week
- ☐ 1 session per week
- ☐ 2 sessions per week
- ☐ 3 sessions per week
- ☐ 4 sessions per week
- ☐ ≥ 5 sessions per week

<u>RECOVERY AND MONITORING</u>

72. Do you believe that performing recovery sessions are beneficial to improving rugby performance?
- ☐ Yes
- ☐ Not sure
- ☐ No

73. Do you implement recovery sessions into your programme?
- ☐ Yes
- ☐ No

74. Please describe why recovery sessions are/ are not implemented.

____

75. Please select all modalities that you prescribe in recovery sessions.
- ☐ Active (aerobic) recovery
- ☐ Cold water immersion
- ☐ Compression garments
- ☐ Contrast temperature water immersion
- ☐ Cryotherapy
- ☐ Foam rolling
- ☐ Massage
- ☐ Stretching
- ☐ Other

76. If you selected 'Other' to question 75, please list other recovery modalities utilized.

____

77. Where do recovery sessions fit into a typical weekly training schedule?

- ☐ Pre-resistance training
- ☐ Post-resistance training
- ☐ Pre-rugby training
- ☐ Post-rugby training
- ☐ Pre-match
- ☐ Post-match
- ☐ Non-training days

78. Do you believe that monitoring athlete wellness (e.g. perceived recovery, mood, stress, muscle soreness, sleep etc.) is beneficial for assessing recovery status in rugby?

- ☐ Yes
- ☐ Not sure
- ☐ No

79. Do you monitor athlete wellness?

- ☐ Yes
- ☐ No

80. Do you monitor menstrual cycle phase?

- ☐ Yes
- ☐ No

81. Do you monitor training and match loads?

- ☐ Yes
- ☐ No

82. How often is athlete wellness monitored?

- ☐ Every session
- ☐ Daily
- ☐ Multiple days / week
- ☐ Weekly
- ☐ Monthly
- ☐ Not monitored

83. Select all sport science technologies used to assist with physical preparation decision making.

- ☐ Actigraphy technologies
- ☐ Biochemical measurement assessment tools
- ☐ Force plates
- ☐ GPS/ accelerometry technologies
- ☐ Heart rate monitoring systems
- ☐ Hydration status assessment tools
- ☐ Isokinetic strength assessment systems

- ☐ Isometric strength assessment systems
- ☐ Jump mats
- ☐ Psychomotor speed assessment tools
- ☐ Radar guns
- ☐ Timing gates
- ☐ Velocity based training systems
- ☐ Other

84. If you selected 'Other' to question 83, please state other technologies utilised.
____

85. Please describe how sport science technologies assist you as a practitioner.
____

UNIQUE ASPECTS OF PHYSICAL PREPARATION IN WOMEN'S RUGBY

86. Please describe any unique aspects of consideration (e.g. contextual, physiological, physical) when working in the physical preparation of women's rugby.
____
